# Magnetic Resonance Imaging Template to Standardize Reporting of Evacuation Disorders

**DOI:** 10.3390/jimaging10120302

**Published:** 2024-11-23

**Authors:** Vittorio Piloni, Tiziana Manisco, Marco Fogante

**Affiliations:** 1 Diagnostic Imaging Center “Diagnostica Marche” Osimo Stazione, 60027 Ancona, Italy; vittorio.piloni@libero.it (V.P.); maniscotiz@live.it (T.M.); 2 Department of Radiology, Maternal-Child, Senological, Cardiological Radiology and Outpatient Ultrasound, University Hospital of Marche, 60126 Ancona, Italy

**Keywords:** MR defecography, static and dynamic pelvic MR, obstructive defecation syndrome, pelvic organ prolapse, radiology reporting, MR defecography template, chronic constipation

## Abstract

Magnetic resonance (MR) defecography, including both static and dynamic phases, is frequently requested by gastroenterologists and colorectal surgeons for planning the treatment of obstructive defecation syndrome and pelvic organ prolapse. However, reports often lack key information needed to guide treatment strategies, making management challenging and, at times, controversial. It has been hypothesized that using structured radiology reports could reduce missing information. In this paper, we present a structured MR defecography template report that includes nine key descriptors of rectal evacuation. The effectiveness and acceptability of this template are currently being assessed in Italy through a national interdisciplinary study.

## 1. Introduction

For radiologists, developing a high-quality report has always been a demanding task, as it is the primary way to convey the results of their diagnostic evaluations to both the referring physician and the patient. Additionally, the report serves as the foundation of communication between researchers and various diagnostic centers.

In simple terms, a report can be defined as a written document, accompanied by appropriate images, that records the medical examination performed by the radiologist, adhering to the quality standards set by scientific societies to determine the presence or absence of disease in individual cases. There are generally two types of reports in use: the free-text report and the structured report, which can be either partially or fully preformatted with essential diagnostic information. Although free-text reports allow the radiologist’s personal style to be better expressed, they are more prone to variability, which can negatively impact the diagnostic content and lead to dissatisfaction among referring physicians.

In the context of evacuation disorders and pelvic floor dysfunctions (ED and PFD), magnetic resonance (MR) with acoustic gel as contrast has largely replaced conventional X-ray techniques and is now considered the method of choice. First described by Lienemann and colleagues in 1997 [1], the subsequent decade saw the production of extensive documentation on the topic of MR proctography (Table 1) to address various aspects, including magnet orientation (whether horizontal or vertical), optimal pulse sequences and fast image acquisition, comparability between MR and X-ray findings, clinical applications, and reference values [2,3,4,5,6,7,8,9,10,11,12,13,14,15,16]. Unfortunately, the issue of reporting received little attention until recently and the reports often omit key information needed for guiding treatment strategies, resulting in management challenges and, at times, controversy. It has been hypothesized that using structured radiology reports can reduce missing information.

To improve the effectiveness of MR defecography reports, we have developed a structured template that includes nine key descriptors designed to best capture the anatomical and dynamic features of various dysfunctions.

The ideal report template, as envisioned by the authors, should be easily understandable and readable by both humans and machines, allowing the data to be integrated into the Radiology Information System and Picture Archiving and Communication System of the local radiology department. In this way, it would facilitate statistical analysis, data exchange between centers, and support research efforts.

## 2. Materials and Methods

An extensive literature search was conducted in the Medline database for articles published between 1991 and 2024 using “MR defecography” and “MRI in pelvic floor dysfunctions” as key terms. The search focused on articles that highlighted the following priorities:

- Defining consistent anatomical landmarks, reference lines, parameters, and easy-to-use measurements;
- Describing diagnostic criteria and grading systems for the most relevant changes in quantitative terms;
- Examining the correspondence between clinical and imaging classification systems for pelvic organ prolapse (POP);
- Evaluating rectal emptying function.

At the end of this work, a structured one-page MR template report was created, designed to be easily comprehensible and readable by both humans and machines. The template includes 9 key descriptors to best characterize various dysfunctions and ensure no relevant data are omitted.

The effectiveness and acceptability of this template are currently being assessed in Italy through a National Interdisciplinary Team Project, involving 13 radiologists and 16 clinicians in a cross-sectional study aimed at promoting better reporting and educational guidance.

## 3. Results

The literature search yielded over 400 papers, which were printed and compiled into three volumes of approximately 250 pages each to facilitate reading and consultation. All studies were reviewed for eligibility, with the highest priority given to:

- Review articles and the most recent multidisciplinary consultation statements.
- Original definitions and descriptions of changes, measurement systems, and grading of POP syndromes.
- Quantification of rectal emptying function.

## 4. Proposed Methodology

### 4.1. Standardized MR Instrumentation, Scanning Technique, and Imaging Protocol

The following description considers the minimum standard requirements, readily available in most settings, to achieve an effective MR defecographic examination. The essential equipment consists of a conventional, horizontally oriented 1.5 T MR scanner and phased-array coils wrapped around the patient’s pelvis connected to the scanner [17].

Regarding the imaging protocol, the following steps have proven to be the most effective in our experience for a successful examination.

#### 4.1.1. Initial Scanning

Begin by scanning the patient along the three body axes before administering rectal contrast (acoustic gel) and with an empty bladder. This will allow for visualization of the entire pelvic anatomy under normal physiological conditions.

#### 4.1.2. Contrast Administration

Slowly administer a standard amount of rectal contrast, up to 240 mL, while continuously monitoring the patient’s sensation of filling. Record both the size of the rectal reservoir and the highest point reached by the contrast column in relation to the spine.

#### 4.1.3. Dynamic Series

Acquire three dynamic, fast series in the midsagittal plane centered over the anorectal junction, with the patient at rest, squeezing, and straining.

#### 4.1.4. Rectal Emptying

Instruct the patient to begin the rectal emptying process at their own pace. Use an acoustic device to mark the start, enabling simultaneous acquisition of images over a full 58-s cycle.

#### 4.1.5. Coronal Series

Repeat the sequence in the coronal plane while the patient expels the remaining rectal contrast.

#### 4.1.6. Axial Imaging of the Levator Hiatus

Image the levator hiatus in the axial plane during its transition from rest to evacuation. Capture this at three horizontal levels (level I: at the midsymphysis; level II: tangent to the inferior border of the symphysis; level III: at the point of maximal descent of the anorectal junction).

#### 4.1.7. Post-Toilet Series

If necessary, acquire a series of images after the patient has finished evacuating.

## 5. Image Analysis

The essential data to include in the report that, in our experience, have been found to be associated with the lowest risk of missing relevant information can be distinguished into anatomical (static MR series) and functional (dynamic MR series).

### 5.1. Static Series

#### 5.1.1. Bony Structures and Landmarks

**Figure 1:**
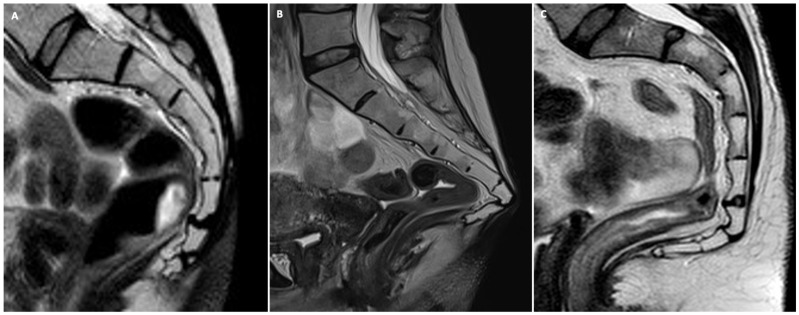
Midsagittal T2-weighted MR images showing the three most common types of sacrococcygeal spine morphology relevant to radiologists when drawing the reference line for measurement of pelvic organs: type 1 (**A**); type 2 (**B**); and type 3 (**C**).

The morphology of the sacrococcygeal spine, whether curved (type 1), squared (type 2), or mixed (type 3), as seen on midsagittal MR T2w images (Figure 1), has relevance when drawing the reference line for the assessment of pelvic organs’ position during the various maneuvers. Any visible congenital abnormality must also be reported.

#### 5.1.2. Endopelvic Fascia and Ligaments

**Figure 2:**
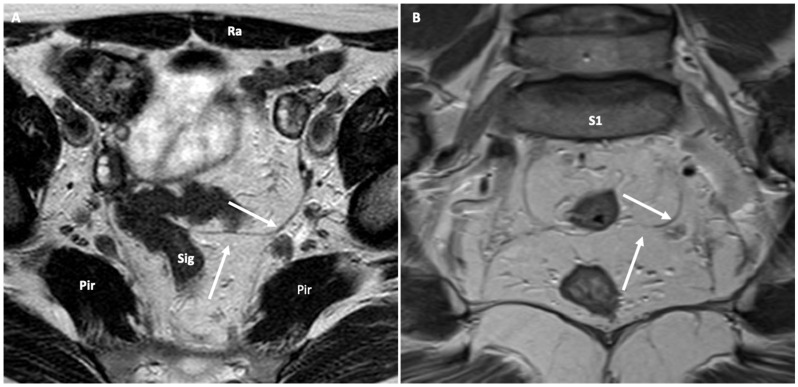
MR appearance of the H-shaped main linear condensation of endopelvic fascia (arrows) as seen on axial (**A**) and coronal (**B**) T2-weighted images to determine anatomical compartments and sub-compartments. Pir: piriformis muscle; Sig: sigmoid colon; Ra: rectum abdominis muscle; S1: 1st sacral bone.

Depending on the scan plane, a few linear condensations of the endopelvic fascia can be identified, which help in defining the subdivision of pelvic compartments and sub-compartments in health and disease. Among all, the main H-shaped fascial subdivision mimicking a scaffold (Figure 2) must be described based on its presence/absence, visibility, and maintained tightness along the entire length without any discontinuity and/or fluttering. Other consistent structures seen include the round, cardinal, and uterosacral ligaments, the sacrospinous and sacrotuberous ligaments, the Waldeyer fascia, the Denonvilliers fascia, mesorectal and presacral fascia, and puboprostatic and rectal ligaments.

#### 5.1.3. Paravaginal and Paraurethral Fascia

Connective tissue containing venous plexus, which is identified as a hyperintense structure surrounding the vaginal wall and female urethra with variable thickness.

#### 5.1.4. Muscles and Fat Recesses

The highest priority is given to the levator ani (LA) muscle, which should be described and displayed separately in its three components: the iliococcygeus, pubococcygeus, and puborectalis muscles. Any discontinuity (tear), avulsion, thickness asymmetry, abnormal signal intensity, and/or fat replacement should be noted in the report. The mean thickness of both sides (calculated as the thickness of the thicker limb plus the thickness of the thinner limb, divided by two) and the degree of asymmetry (calculated as the thickness of the thicker limb divided by the thickness of the thinner limb) should be measured. The thresholds not to be exceeded are 4.4 ± 1.6 mm for thickness and 1.4 ± 0.5 mm for asymmetry, respectively [18]. Due to their frequent involvement in pelvic floor dysfunctions, the same considerations apply to the piriformis muscle, obturator muscle, and transverse perineum muscle.

#### 5.1.5. Neural Pathways

Any overt distortion, displacement, or entrapment occurring along the course of the lumbosacral plexus and pudendal nerve, namely the ischiatic foramen and Alcock’s canal, should be included in the report as a potential source of chronic pelvic pain syndromes.

#### 5.1.6. Visceral Components

**Figure 3:**
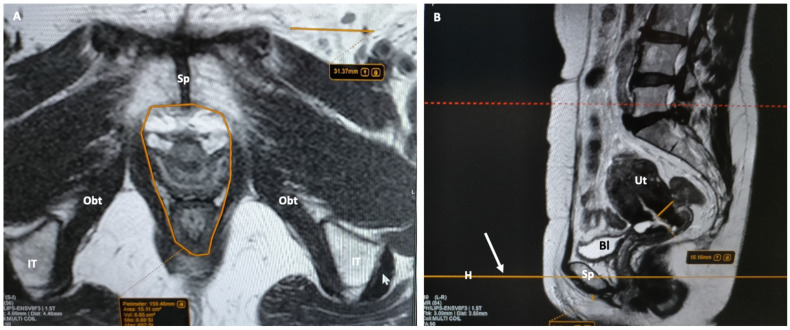
Method for free-hand tracing the area (orange contour) of levator ani hiatus on axial T2-weighted MR image (**A**) by selecting the scan plane on midsagittal image (**B**) and taking the middle of the long axis of pubic bone as reference (arrow). Sp: symphysis pubis; IT: ischial tuberosity; Obt: obturator muscle; Bl: bladder; Ut: uterus; H orange line: horizontal scan plane corresponding to (**A**).

The urogenital hiatus, the space included within the medial margins of the levator ani muscle [19,20], must be described on axial T2w images for its size and content. The antero-posterior diameter (distance from the pubic symphysis to the ventral margin of the puborectalis sling), the transverse diameter (distance between the medial borders of the levator ani muscles), and the area should be measured and expressed in millimeters and square centimeters, respectively. From front to back, the anatomical structures to describe include the female urethra, vaginal canal, and the anal sphincter complex (Figure 3).

**Figure 4:**
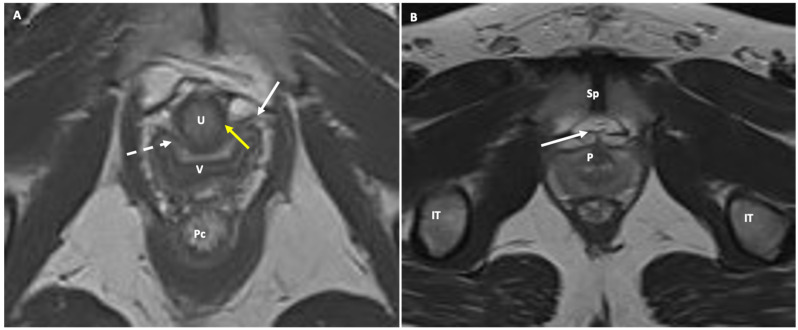
Axial T2-weighted MR images of levator ani hiatus showing the periurethral (white arrow), the paraurthral (yellow arrow), and pubourethral (white dotted line) female ligaments (**A**). Corresponding similar structures can occasionally be seen in young males (**B**) in the retropubic space just ventral to the prostate (arrow). U: urethra; V: vagina; Pc: pubococcygeal muscle; Sp: symphysis pubis; IT: ischial tuberosity; P: prostate.

- Female urethra: This appears as a target-like structure, showing a central hypointense inner ring, which indicates the muscularis mucosa. Surrounding this is a middle, thicker ring of intermediate signal intensity, resulting from a combination of submucosa, longitudinal, and circular smooth muscle. The third, thinner hypointense outer ring represents the striated sphincter muscle. The surrounding space shows high signal intensity, representing connective tissue and smooth muscle within a highly collagenized vascular matrix. Within this space, three distinct ligaments can be identified at specific points along the urethra. At the 30th percentile, the periurethral ligament is visible as a thin hypointense linear structure originating from the medial aspect of the puborectal muscle, running ventrally to the urethra. The paraurethral ligament appears as a slightly oblique hypointense structure connecting the lateral wall of the urethra to the periurethral ligaments (Figure 4). At the 50th percentile, the pubourethral ligament is visible as a hypointense structure connecting the lateral aspect of the urethra to the arcus tendineus fasciae pelvis. In young males, corresponding structures may occasionally be visible in the retropubic space, just anterior to the prostate apex. On sagittal T2-weighted MR, the urethra appears as a cylindrical structure extending from the bladder neck to the external meatus. The signal intensity mirrors that described above, with the internal meatus as the zero point and the external meatus at the 100th percentile.
- Vaginal canal: From internal to external, the vaginal canal shows a high-signal intensity (due to mucous or secretions) surrounded by three layers with differing signal intensities: a low-signal-intensity inner layer corresponding to squamous keratinized epithelium and lamina propria; an intermediate-signal-intensity middle layer representing the muscular wall; and a low-signal-intensity outer layer representing loose connective tissue with its vascular supply.
- Anal sphincter complex: This shows variable composition and signal intensity on axial T2-weighted images depending on the level. The upper part consists of the internal sphincter, longitudinal muscle, and puborectal muscle. The middle part features the intersphincteric space, which appears as a high-signal-intensity, slit-like space between the internal sphincter (5 mm thick, intermediate signal intensity) and the external sphincter (1.5 mm thick, low signal intensity). A thin hypointense circular structure within this space represents the longitudinal muscle, which is a continuation of the outer longitudinal smooth muscle of the rectum. The lower part contains the external sphincter and the longitudinal muscle layer. On mid-coronal T2-weighted images, the thicker inner layer is formed by the two halves of the internal sphincter in apposition, while the intersphincteric space is visible as a thin, high-signal-intensity layer. At the outer margin, a cleft divides the puborectalis muscle (above) from the external sphincter (below). On midsagittal T2-weighted MR images, the anal sphincter appears as a low-signal-intensity, homogeneous, cylindrical structure, approximately 4 cm long, composed of muscle layers extending from the attachment of the levator ani muscle to the rectum. Lastly, the perianal spaces are clearly seen as two symmetrical, high-signal-intensity, fat-containing, pyramid-shaped spaces surrounding the hypointense anal canal, with multiple fibrous septa. The apex is visible at the origin of the V-shaped levator ani muscle, along with the supralevator space above it.

### 5.2. Dynamic Series

#### 5.2.1. Rectal Diameters at Capacity

This parameter characterizes the so-called “rectal compliance”, i.e., the ability of the rectal reservoir to adapt its shape and size to the volume of intraluminal content. Together with the ability to retain the contrast injected without involuntary loss [21], both are considered useful criteria in the differential diagnosis of fecal retention and incontinence, respectively.

#### 5.2.2. Vesicourethral Angle

This angle is determined on sagittal MR images by the intersection of a line drawn through the long axis of the urethra with a line parallel to the bladder base. The reported value is 122° ± 11 in normal subjects compared to 148° ± 13° in those with stress urinary incontinence.

#### 5.2.3. The Length of Retropubic Urethra (UL)

It is measured from the bladder neck to the intersection with a line drawn at the inferior edge of the pubic bone; the portion below where the urethra describes a curve to reach the external meatus is called curved urethra. According to the method of deSouza [22], the ratio of retropubic UL to its total length should be no less than 82.6% ± 7.4 in normal subjects compared to 57.4% ± 9.8 (SD) of patients with stress urinary incontinence.

#### 5.2.4. The Posterior Anorectal Angle (ARA)

It is defined as the intersection between the central axis of the anal canal with the line drawn tangent to the lower third of the rectal floor. For normal and abnormal values, the reader is kindly referred to the Joint Recommendations and the Consensus Meeting Proceedings published by El Sayed et al. and Gourland et al. [23,24].

#### 5.2.5. The Pubococcygeal Line (PCL)

This is defined as a line drawn from the lower border of posterior symphysis pubis to the last sacrococcygeal joint. Despite some drawbacks, as in the case of congenital abnormalities of the sacrococcygeal spine, the PCL is the most widely shared reference line used all over the world [25] to assess the position of pelvic organs in the pelvis at rest, squeeze, strain, and evacuation. As an alternative, the horizontal line (HL) drawn tangent to the lowermost border of the symphysis pubis can also be considered in clinical practice.

#### 5.2.6. The HMO Classification System

**Figure 5:**
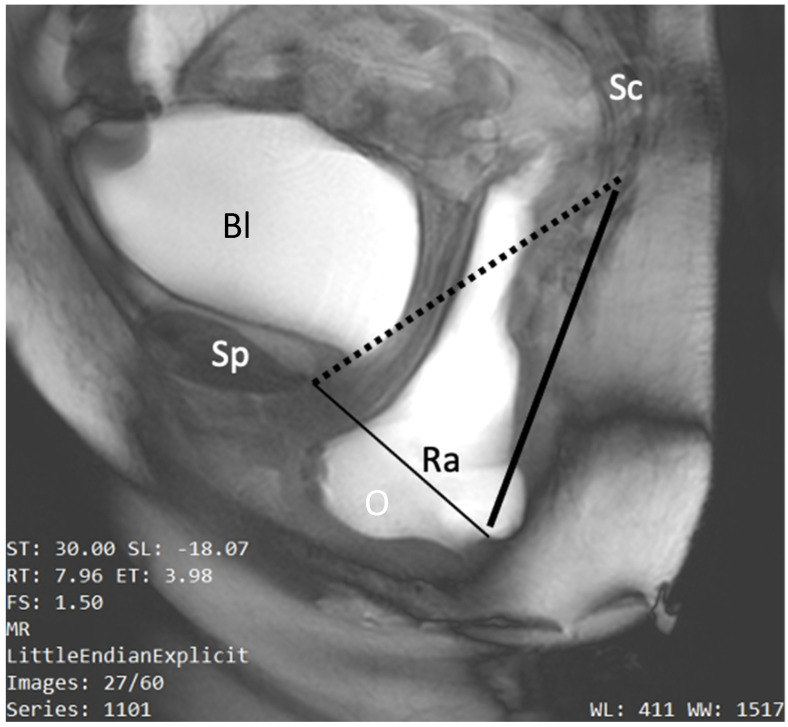
Dynamic midsagittal MR image taken during evacuation for quantification of pelvic organ descent by the HMO system according to Comiter. Pubococcygeal reference line (dotted line); H-line which measures the distance of anorectal junction to the pubic bone as index of levator hiatus width (thin continuous line); M-line indicating the length of levator plate muscle (thick continuous line). O: descent of the bladder and rectum relative to the PCL reference line; Sp: symphysis pubis–Bl: bladder; Ra: rectal ampulla; Sc: sacrococcygeal spine.

This is the acronym for urogenital hiatus (H), levator plate muscle (M), and organs (O), respectively, which is the unsurpassed method described in the radiologic literature since 1999 by Comiter et al. [5] to classify the degree of pelvic organ prolapse syndromes relative to the PCL (Figure 5). More precisely, the H-line (average 7.5 ± 1.5 cm in prolapse group vs. 5.2 ± 1.8 cm in normal subjects) measures the distance from the posterior lowermost border of the pubis to the posterior anorectal junction; the M-line (4.1 ± 1.5 cm in prolapse group vs. 1.9 ± 1.2 cm in normal subjects) measures the descent of the levator plate muscle from the PCL; and the O-line characterizes the degree of visceral prolapse beyond the H-line.

#### 5.2.7. Morphologic Changes on Evacuation

Anterior compartment:

- Bladder neck hypermobility: defined as an abnormal descent during straining, with the bladder neck descending more than 1 cm below the pubis, with or without funneling (shortening and dilation of the proximal portion). It is associated with a horizontal orientation and a urethrovescical (UV) angle greater than 115°.
- Cystocele: defined as a focal protrusion of the bladder profile outside the pelvic cavity, with the bladder base located below the pubic symphysis or more than 1 cm below the pubococcygeal line (PCL).

Middle compartment:

- Vaginal vault and cervical prolapse: defined as an abnormal descent and eversion of the vaginal vault or cervix to less than 1 cm above the PCL.
- Cul-de-Sac herniation: defined as an abnormal deepening of the pelvic peritoneum beyond the normal confines of the rectovaginal space or widening of the rectovaginal space containing fat, small bowel, or sigmoid colon.

Posterior compartment:

- Rectocele: defined as an anterior protrusion of the rectal wall during evacuation, greater than 2 cm beyond the expected margin of a line extending upward from the anterior margin of the anal canal.
- Intussusception: defined as a full-thickness circumferential rectal wall invagination greater than 3 mm in thickness, descending toward the anal canal during evacuation but not extending beyond the anal verge. It is seen as a funnel-shaped infolding of the upper rectal wall, creating a “tube within a tube”. The thickness of the intraluminal defect, the depth of descent, and the distance from the point of inversion to the anal verge are measured.
- Mucous rectal prolapse: defined as an intraluminal infolding of the rectal wall during evacuation, less than 3 cm in thickness.
- External rectal prolapse: defined as a full-thickness protrusion of the rectal wall, occurring either at rest, during straining, or during evacuation. It may be reduced spontaneously or manually.
- Anal gaping: defined as a lack of apposition of the anal walls, allowing intrarectal contrast to penetrate the proximal half of the anal canal without leakage.
- Incontinence: defined as the loss of contrast material before reaching maximum rectal capacity, associated with an inability to interrupt the stream by voluntary contraction of the anal sphincters (poor stop test).
- Dyssynergia: defined as transient or persistent paradoxical contraction of either the puborectalis muscle or the anal sphincters during evacuation. This is seen as a persistent impression at the posterior margin of the anorectal junction, along with a lack of anorectal angle (ARA) widening and a failure of anal canal widening.
- Pelvic organ descent: defined as the difference between the resting and straining positions of the bladder neck, prostate base, vaginal vault, and anorectal junction (ARJ). This descent is measured relative to a reference line (PCL or horizontal line) and indicated by a number preceded by a minus sign (−) for above or a plus sign (+) for below the reference. Normal values are −3 cm for the bladder base, prostate, and vaginal vault and −2 cm for the ARJ.

### 5.3. The Emptying Pattern

The speed and completeness of rectal emptying, the number of attempts employed to expel the contrast, the total amount retained, and the site of entrapment all contribute to defining the so-called “rectal clearance”, a parameter which has recently been described to effectively distinguish patients with fecal obstruction (average clearance 31.18 ± 23.34 cm^2^) from those with incontinence (58.27 ± 16.50 cm^2^) and pelvic organ prolapse (45.12 ± 22.53 cm^2^) [26].

More specifically, according to the original method described by Narayanan SP et al. [27], the clearance of acoustic gel during rectal emptying is calculated planimetrically on MR sagittal images as the value of rectal area before evacuation minus the value after evacuation divided by the value before evacuation × 100. Despite the high standard deviation, average values of rectal clearance differed significantly (*p* < 0.001) among the three groups, indicating that the application of the method allows a better discrimination of categories and subcategories of pathologies in patients with functional disorders of defecation, with potential influence on therapy planning.

## 6. The Proposed MR Template

**Figure 6:**
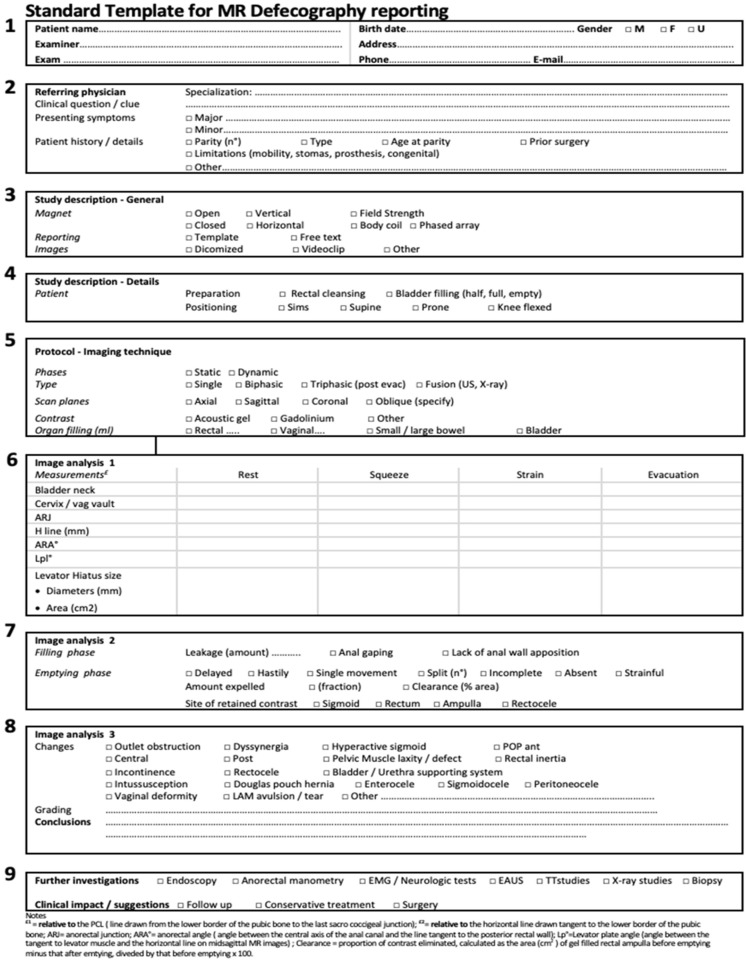
Nine key descriptors of the structured MR template report to use in MR defecography.

All the above has been synthesized by us into a one-page reporting template (Figure 6), which contains information on patient identification, institution, the referring physician, instrumentation, and imaging protocol details. It also includes a description of the main presenting symptoms and any prior surgeries. At the end, there is a space for diagnostic conclusions and recommendations for further investigation, if necessary.

More specifically, the template includes the following components: the first section contains the identifying data of the institute, examination type, performing examiner, and patient details; the second section includes the referring physician’s specialization, the clinical question, presenting symptoms, and any additional relevant patient details, such as limiting factors; the third section, “study description”, specifies the general characteristics of the equipment, imaging protocol, and exported images, followed by a more specific sub-section; the fourth section covers patient preparation and positioning; the fifth section describes the phases of the examination, scan planes, administered contrast, and organ filling; the sixth section analyzes images during both the static and dynamic phases, including measurements of various parameters; the seventh section contains information about filling capacity, the position of pelvic organs by compartment, and details of emptying; the eighth section covers observed changes with their grading and the diagnostic conclusions; the ninth and final section, which is arguably the most important, contains the examiner’s opinion and suggestions for further investigations, whether imaging or non-imaging studies, and the potential clinical impact of the examination on therapeutic decisions, whether medical or surgical.

Explanations of acronyms, chosen parameters, reference landmarks, and related references are provided in the footnotes below the form. Additionally, to further improve interaction between physicians and radiologists, a 1–5 rating scale (with 1 = extremely poor and 5 = excellent) has been developed. This scale is intended to quantitatively assess the quality of the request (Table 2), aiming to establish a minimum standard for effective co-operation between the referring physician and the radiologist.

The goal of the radiologist’s report is to help clinicians address the following questions: why does the referred patient suffer from urinary/fecal incontinence or obstruction syndrome? Is the prolapse observed during physical examination an isolated issue or part of a more complex descending perineum syndrome? Why is there a recurrence of prolapse after an apparently successful surgical repair?

To answer these questions, full co-operation with the referring physician is critical, along with proper patient preparation. The patient must be informed about the phases and objectives of the examination. The radiologist should also maintain a logical and structured approach in reporting, focusing on the essential observations and distinguishing between anatomical features (from the static series) and functional ones (from the dynamic series) observed across the three pelvic compartments.

The proposed template aims to improve the characterization of pelvic floor dysfunctions on imaging. It is currently undergoing validation in Italy by a Joint Interdisciplinary Committee with the goal of extending its use nationwide.

## 7. Conclusions

The management of evacuation disorders and pelvic organ prolapse can be challenging for both gastroenterologists and colorectal surgeons. In most cases, due to the multifactorial causes involved and the complexity of symptom combinations, a thorough characterization of the dysfunction through clinical examination and imaging is crucial for selecting the most appropriate treatment.

MR defecography has become the preferred imaging modality, offering an unparalleled ability to display both anatomical and functional features in detail. It is often referred to as the “All-in-One examination”. However, the diagnostic yield is sometimes not fully characterized by radiologists in their final reports, mainly due to excessive variability in the description and classification of findings, which complicates decision-making processes. The use of a structured radiology report, or template, as described in this article, could improve the characterization, management, and follow-up of patients.

## Figures and Tables

**Table 1:** Top list of the 15 articles published on MR proctography by year and category.

N°	First Author	Year	Journal	Reference List (n°)	Category
1	Healy JC	1997	AJR	[2]	MR/X-Ray comparison
2	Maglinte DD	1999	AJR	[3]	Defects association
3	Gufler H	1999	J Mag Res I	[4]	Fast Pulse sequence
4	Comiter CV	1999	J Urol	[5]	Grading system
5	Vanbeckevoort D	1999	J Mag Res I	[6]	MR/X-Ray comparison
6	Kelvin FM	2000	AJR	[7]	MR/X-Ray comparison
7	Goh H	2000	AJR	[8]	Asymptomatic subjects
8	Pannu HK	2000	Radiographics	[9]	Changes in POP
9	Singh K	2001	Am J Obstet Gynecol	[10]	Grading system
10	Tunn R	2001	Obstet Gynecol	[11]	MR pelvic floor anatomy
11	Roos JE	2002	Radiographics	[12]	Vertical magnet
12	Bertshinger KM	2002	Radiology	[13]	Vertical magnet
13	Fielding JR	2002	Radiographics	[14]	Reference values in POP
14	Cortes E	2004	Obstet Gynecol	[15]	Reference values in POP
15	Dvorkin LS	2004	Colorectal Disease	[16]	MR/X-Ray comparison

**Table 2:** Rating scale for quality of the examination requests.

Score	Grading	Description
0	To be rejected	Wrong, misleading content
1	Extremely poor	No > written notice of the examination
2	Poor	As 1 + patient complaint
3	Mediocre	As 2 + list of presenting symptoms
4	Good	As 3 + physical examination details
5	Excellent	As 4 + history, original records, clinical question

## Data Availability

Dataset available on request from the authors.

## References

[1] Lienemann A., Anthuber C., Baron A., Kohz P., Reiser M. (1997). Dynamic MR colpocystorectography a ssessing pelvic flooor descent. Eur. Radiol..

[2] Healy J.C., Halligan S., Reznek R.H., Watson S., Bartram C.I., Phillips R., Armstrong P. (1997). Dynamic MR imaging compared with evacuation proctography when evaluating anorectal configuration and pelvic floor movement. Am. J. Roentgenol..

[3] Maglinte D.D., Kelvin F.M., Fitzgerald K., Hale D.S., Benson J.T. (1999). Association of compartment defects in pelvic floor dys-function. Am. J. Roentgenol..

[4] Gufler H., Laubengerger J., DeGregorio G., Dochnicht S., Langer M. (1999). Pelvic floor decent: Dynamic MR imaging using a half.fourier RARE sequence. J. Magn. Reson. Imaging.

[5] Comiter C.V., Vasavada S.P., Barbaric Z.L., Gousse A.E., Raz S. (1999). Grading pelvic prolapse and pelvic floor relaxation using dynamic magnetic resonance imaging. Urology.

[6] Vanbeckevoort D., Van Hoe L., Oyen R., Ponette E., De Ridder D., Deprest J. (1999). Pelvic floor descent in females: Comparative study of colpocystodefecography and fast MR imaging. J. Magn. Reson. Imaging.

[7] Kelvin F.M., Maglinte D.D.T., Hale D.S., Benson J.T. (2000). Female pelvic organ prolapse: A comparison of triphasic dynamic MR imaging and triphasic fluoroscopic cystocoloproctography. Am. J. Roentgenol..

[8] Goh V., Halligan S., Kaplan G., Healy J.C., Bartram C.I. (2000). Dynamic MR imaging of the pelvic floor in asymptomatic subjects. Am. J. Roentgenol..

[9] Pannu H.K., Kaufman H.S., Cundiff G.W., Genadry R., Bluemke D.A., Fishman E.K. (2000). Dynamic MR imaging of pelvic organ prolapse: Spectrum of abnormalities. RadioGraphics.

[10] Singh K., Reid W.M., Berger L.A. (2001). Assessment and grading of pelvic organ prolapse by use of dynamic resonance imaging. Am. J. Obstet. Gynecol..

[11] Tunn R., DeLancey J.O., Quint E.E. (2001). Visibility of pelvic organ support system structures in magnetic images without an endovaginal coil. Am. J. Obstet. Gynecol..

[12] Roos J.E., Weishaupt D., Wildermuth S., Willmann J.K., Marincek B., Hilfiker P.R. (2002). Experience of 4 years with open MR defecography: Pictorial review of anorectal anatomy and disease. RadioGraphics.

[13] Bertschinger K.M., Hetzer F.H., Roos J.E., Treiber K., Marincek B., Hilfiker P.R. (2002). Dynamic MR imaging of the pelvic floor performed with patient sitting in an open-magnet unit versus with patient supine in a closed-magnet unit. Radiology.

[14] Fielding J.R. (2002). Practical MR imaging of female pelvic floor weakness. Radiographics.

[15] Cortes E., Reid W.M.N., Singh K., Berger L. (2004). Clinical examination and dynamic magnetic resonance imaging in vaginal prolapse. Obstet. Gynecol..

[16] Dvorkin L.S., Hetzer F., Scott S.M., Williams N.S., Gedroyc W., Lunniss P.J. (2004). Open-magnet MR defaecography compared with evacuation proctography in the diagnosis and management of patients with rectal intussuception. Color. Dis..

[17] Piloni V., Vernelli M., Civitella V., Giomo G., Felici T. (2021). MR-defecography: Which protocol is most appropriate?. EC Gastroenterol. Dig. Syst..

[18] Kim J.K., Kim Y.J., Choo M.S., Cho K.-S. (2003). The urethra and its supporting structures in women with stress urinary incontinence: MR imaging using an endovaginal coil. Am. J. Roentgenol..

[19] DeLancey J.O., Hurd W.W. (1998). Size of the urogenital hiatus in the levator ani muscles in normal women and women with pelvic organ prolapse. Obstet. Gynecol..

[20] Piloni V., Bergamasco M., Chiapperin A. (2019). Quantification of levator ani hiatus enlargement by magnetic resonance imaging in males and females with pelvic organ prolapse. J. Vis. Exp..

[21] Piloni V., Pignalosa F., Lucchetti N., Felici T., Andreatini J., Pillon E. (2019). Biphasic MRI examination of fecal incontinence with external coil: Technique and clinical applications. EC Gastroenterol. Dig. Syst..

[22] Desouza N.M., Daniels O.J., Williams A.D., Gilderdale D.J., Abel P.D. (2002). female urinary genuine stress incontinence: Anatomic considerations at MR imaging of the paravaginal fascia and urethra—Initial observations. Radiology.

[23] El Sayed R.F., Alt C.D., Maccioni F., Meissnitzer M., Masselli G., Manganaro L., Vinci V., Weishaupt D. (2016). Magnetic resonance imaging of pelvic floor dysfunction—Joint recommendations of the ESUR and ESGAR pelvic floor working group. Eur. Radiol..

[24] Gurland B.H., Khatri G., Ram R., Hull T.L., Kocjancic E., Quiroz L.H., El Sayed R.F.E., Jambhekar K.R., Chernyak V., Paspulati R.M. (2021). Consensus definitions and inter-pretation templates for magnetic resonance imaging of defecatory pelvic floor disorders: Proceedings of the Consensus Meeting of the Pelvic Floor Disorders Consortium of the American Society of Colon and rectal Surgeons, the Society of Abdominal Radiology, the International Continence Society, the American Urogynecologic Society, the International Urogynecological Association, and the Society of Gynecologic Surgeons. Am. J. Roentgenol..

[25] Broekhuis S.R., Futterer J.J., Barentsz J.O., Vierhout M.E., Kluivers K.B. (2009). A systematic review of clinical studies on dynamic magnetic resonane imaging of pelvic organ prolapse: The use of reference lines and anatomical landmarks. Int. Urogynecol. J..

[26] Piloni V., Fogante M., Manisco T. (2022). Advances in MR defecography: Analysis of rectal clearance. EC Gastroenterol. Dig. Syst..

[27] Narayanan S.P., Sharma M., Fletcher J.G., Karwoski R.A., Holmes D.R., Bharucha A.E. (2019). Comparison of changes in rectal area and volume during MR evacuation proctography in healthy and constipated adults. Neurogastroenterol. Motil..

